# Free-living marine nematodes from San Antonio Bay (Río Negro, Argentina)

**DOI:** 10.3897/zookeys.574.7222

**Published:** 2016-03-28

**Authors:** Gabriela Villares, Virginia Lo Russo, Catalina Pastor de Ward, Viviana Milano, Lidia Miyashiro, Renato Mazzanti

**Affiliations:** 1 Laboratorio de Meiobentos LAMEIMA-CENPAT-CONICET, Boulevard Brown 2915, U9120ACF, Puerto Madryn, Argentina; 2 Universidad Nacional de la Patagonia San Juan Bosco, sede Puerto Madryn. Boulevard Brown 3051, U9120ACF, Puerto Madryn, Argentina; 3 Centro de Cómputos CENPAT-CONICET, Boulevard Brown 2915, U9120ACF, Puerto Madryn, Argentina

**Keywords:** Nematoda, Enoplea, Chromadorea, South Atlantic

## Abstract

The dataset of free-living marine nematodes of San Antonio Bay is based on sediment samples collected in February 2009 during doctoral theses funded by CONICET grants. A total of 36 samples has been taken at three locations in the San Antonio Bay, Santa Cruz Province, Argentina on the coastal littoral at three tidal levels. This presents a unique and important collection for benthic biodiversity assessment of Patagonian nematodes as this area remains one of the least known regions. In total 7,743 specimens of free-living marine nematodes belonging to two classes, eight orders, 37 families, 94 genera and 104 species were collected.

## Introduction

**Figure 1.**
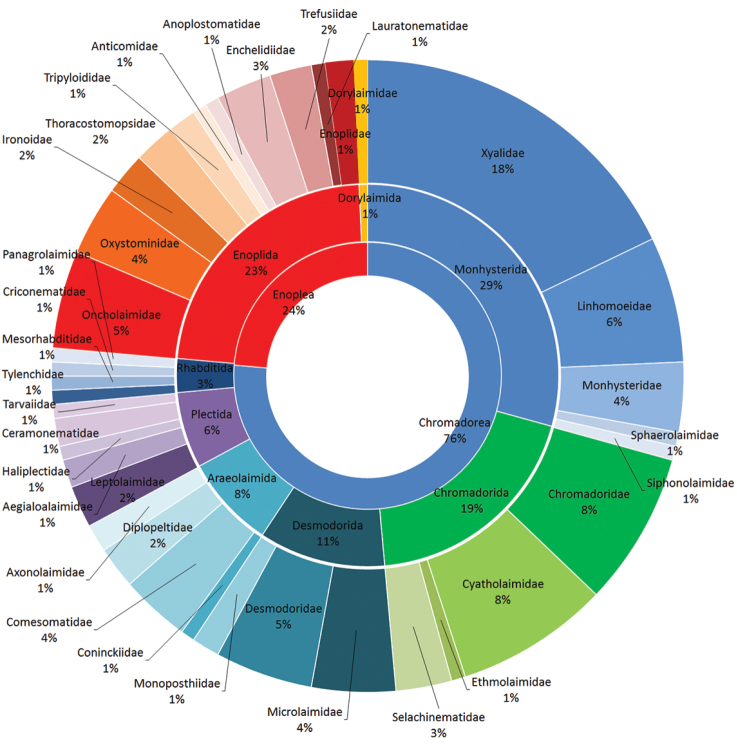
Taxonomic coverage by class, order and family.

This is the first study on nematodes performed on a sub-Antarctic salt marsh along the coast of Río Negro Province, Argentina. This site has a high biodiversity and was declared a Protected Natural Area N°2.670 of the province of Río Negro in 1993 as well as an international reserve of the hemispheric network of Shorebird Reserve within the Wetlands program for the Americas. However, it is also an urban center where economic, industrial and tourist activities take place. The objectives of the study were to collect, identify and discover the structure and diversity of the nematode community of San Antonio Bay. The coverage (Figure 1) of this dataset includes two classes: Chromadorea (76%) and Enoplea (24%); eight orders: Monhysterida (39%), followed by Enoplida (23%) and Chromadorida (19%) as main occurrences; and thirty-seven families (see Figure 1).

## Taxonomic ranks

**Kingdom**: Animalia

**Phylum**: Nematoda

**Class**: Chromadorea, Enoplea

**Order**: Monhysterida, Enoplida, Chromadorida, Desmodorida, Araeolaimida, Plectida, Rhabditida, Dorylaimida.

**Family**: Xyalidae, Linhomoeidae, Monhysteridae, Sphaerolaimidae, Siphonolaimidae, Chromadoridae, Cyatholaimidae, Ethmolaimidae, Selachinematidae, Microlaimidae, Desmodoridae, Monoposthiidae, Coninckiidae, Comesomatidae, Diplopeltidae, Axonolaimidae, Leptolaimidae, Aegialoalaimidae, Haliplectidae, Ceramonematidae, Tarvaiidae, Tylenchidae, Mesorhabditidae, Criconematidae, Panagrolaimidae, Oncholaimidae, Oxystominidae, Ironoidae, Thoracostomopsidae, Tripyloididae, Anticomidae, Anoplostomatidae, Enchelidiidae, Trefusiidae, Lauratonematidae, Enoplidae, Dorylaimidae.

**Genera**: *Odontophora*, *Synodontium*, *Comesoma*, *Metasabatieria*, *Sabatieria*, *Coninckia*, *Campylaimus*, *Diplopeltula*, *Chromadora*, *Chromadorella*, *Chromadorina*, *Prochromadorella*, *Actinonema*, *Rhips*, *Dichromadora*, *Neochromadora*, *Spilophorella*, *Marylynnia*, *Paracantonchus*, *Paracyatholaimus*, *Pomponema*, *Paraethmolaimus*, *Gammanema*, *Halichoanolaimus*, *Latronema*, *Molgolaimus*, *Metachromadora*, *Onyx*, *Polysigma*, *Spirinia*, *Bolbolaimus*, *Microlaimus*, *Nudora*, *Desmolaimus*, *Metalinhomoeus*, *Terschellingia*, *Eleutherolaimus*, *Paralinhomoeus*, *Siphonolaimus*, *Diplolaimella*, *Diplolaimelloides*, *Halomonhystera*, *Monhystera*, *Sphaerolaimus*, *Amphimonhystera*, *Cobbia*, *Daptonema*, *Gonionchus*, *Linhystera*, *Metadesmolaimus*, *Omicronema*, *Paramonohystera*, *Promonhystera*, *Pseudosteineria*, *Rhynchonema*, *Theristus*, *Xyalidae* gen.1, *Ceramonema*, *Pselionema*, *Tarvaia*, *Haliplectus*, *Cyartonema*, *Deontolaimus*, *Leptolaimus*, *Mesorhabditis*, *Macroposthonia*, *Panagrolaimus*, *Tylenchus*, *Dorylaimus*, *Chaetonema*, *Cephalanticoma*, *Enoplus*, *Epacanthion*, *Oxyonchus*, *Thoracostomopsidae* gen.1, *Conilia*, *Dolicholaimus*, *Syringolaimus*, *Halalaimus*, *Calyptronema*, *Eurystomina*, *Abelbolla*, *Adoncholaimus*, *Viscosia*, *Metoncholaimus*, *Oncholaimus*, *Lauratonema*, *Rhabdocoma*, *Trefusia*, *Trefusiidae* gen.1, *Bathylaimus*, *Tripyloides*.

**Species with higher occurrences**: *Microlaimus globiceps*, *Paraethmolaimus dahli*, *Thalassomonhystera parva*, *Microlaimus decoratus*, *Diplolaimelloides oschei*, *Nudora crepidata*, *Viscosia macramphida*, *Chromadorina longispiculum*, *Diplolaimella gerlachi*, *Leptolaimus luridus*.

## Spatial coverage

**Figure 2.**
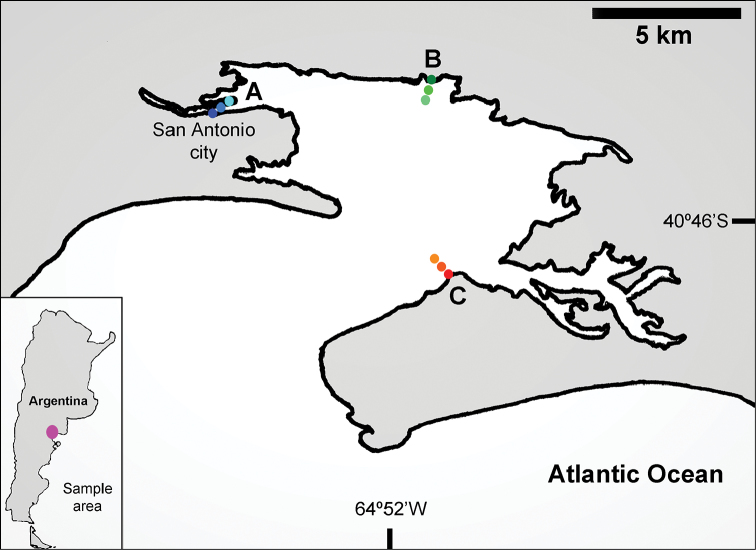
Spatial coverage. San Antonio Bay, Argentina. Sites: **A** “Ciudad” **B** “Baliza Camino” **C** “Banco Perdices”. Levels = u, m, l..

**Figure 3.**
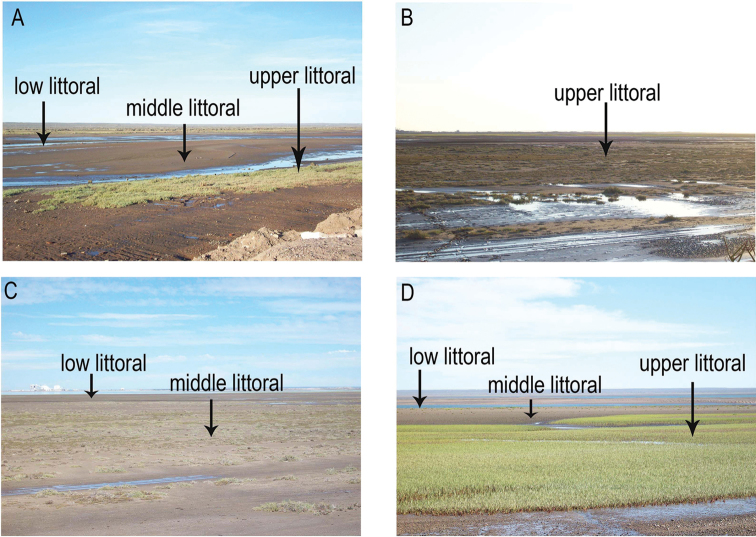
San Antonio Bay, Argentina. Views from the sampling sites. **A** “Ciudad” **B, C** “Baliza Camino” **D** “Banco Perdices”.

**General spatial coverage**: San Antonio Bay, Río Negro Province, Argentina (Figure 2). For this study three sites were selected: “Ciudad” (A), located in the north of the bay; “Baliza Camino” (B), located off the mouth of the bay of San Antonio and “Banco Perdices” (C), located 12 km south of “Las Grutas”. At each sampling site, three tidal levels were chosen: upper-littoral, high tide, salt-marsh habitat (u); middle littoral, mean tide, un-vegetated habitat (m) and low littoral, low tide, un-vegetated habitat (l) (Figure 3).

**Coordinates**: “Ciudad”: Au = 40°43'40.2"S; 64°57'41.1"W; Am = 40°43'39.0"S; 64°57'41.6"W; Al = 40°43'39.0"S; 64°57'39.5"W. “Baliza Camino”: Bu = 40°42'59.9"S; 64°50'46.8"W; Bm = 40°43'05.8"S; 64°50'58.5"W; Bl = 40°43'11.6"S; 64°51'14,6"W. “Banco Perdices”: Cu = 40°47'00.8"S; 64°50'54.3"W; Cm = 40°47'05.6"S; 64°51'17.8"W; Cl = 40°46'51.9"S; 64°51'02.8"W.

## Temporal coverage

12–14 February 2009.

## Methods

**Sampling description**: At each site and level location, four replicates (20 ml) were sampled with a PVC syringe (60 ml, inner diameter 2.9 cm) and separated by a distance of 5–10 m each: four for marine nematodes counts, two for organic matter and two for sediment analyses. Each sample was fixed in situ, with a solution of 5% formaldehyde in filtered sea water with the addition of Rose Bengal tint. Marine nematodes were extracted from samples using the elutriation/decantation LUDOX TM (colloidal silica polymer) method at a specific gravity of 1.15, quantifying only organisms passing through a 500 µm mesh and then retained by a 63 µm mesh. Samples were evaporated to anhydrous glycerol and permanent slides made (Somerfield and Warwick 1996). The taxonomic classification followed proposed by De Ley and Blaxter (2004). For the identification of species international keys (Platt and Warwick 1983, Platt and Warwick 1988, Warwick et al. 1998, Lorenzen 1994, Abebe et al. 2006) and taxonomical papers (Pastor de Ward 1978, 1980, 1984a, b, c, d, e, 1985, 1986, 1988, 1989, 1990, 1991, 1993, 1995a, b, 1996, 1998a, b, c, 1999, Pastor de Ward and Lo Russo 2009, Villares and Pastor de Ward 2012, Lo Russo et. al. 2012, Villares et al. 2013, Pastor de Ward et al. 2013, Lo Russo et. al. 2015) were used. Holotypes and paratypes are deposited in the Collection of Nematodes of the Centro Nacional Patagónico (CENPAT-CONICET), Chubut, Argentina recognized as National Service of Biological Data of Argentina and included in their web page from 2011 (http://www.gbif.org/dataset/06df03fc-8973-490c-af74-089fffae9e24; http://www.gbif.org/dataset/d592283b-b00e-4a39-9499-289842ccddf1).

## Project details

**Project title**: “*Comparación de comunidades de nematodos de marismas de San Antonio Oeste (río Negro) y San Julián (Sta. Cruz*)”. [Comparison of nematode marsh communities of San Antonio Oeste (Río Negro) and San Julián (Sta. Cruz)]. Doctoral thesis Universidad Nacional del Comahue (Lo Russo 2012).

“*Diversidad funcional y producción secundaria de las comunidades de nematodos de las marismas de San Antonio (Río Negro) y de la ría de San Julián (Santa Cruz*)”. [Functional diversity and secondary production of nematode marsh communities of San Antonio (Río Negro) estuary and San Julián (Santa Cruz)”. Doctoral thesis Universidad Nacional del Comahue (Villares 2014).

**Personnel**: Catalina Pastor de Ward (Project Director, meio-benthos specialist), Virginia Lo Russo and Gabriela Villares (field work, nematodes identification, data collection and analysis), Viviana Milano (grant-holding student, data input), Lidia Miyashiro (Darwin core data input), Renato Mazzanti (software engineer, data base manager).

**Study extent description**: The San Antonio Bay marine nematodes is a dataset that gives new insights on the taxonomic and geographic distribution of south Atlantic marine nematodes, covering an under-explored region of the southern Atlantic coasts. This is the first study on marine nematodes in this site. This dataset presents species occurrences and species richness of the individual free-living marine nematodes present at three coastal areas (“Ciudad”, “Baliza Camino”, “Banco Perdices”) of the San Antonio Bay at three different tidal levels (upper, middle and low-littoral).

In total 7,443 specimens of free-living marine nematodes belonging to two classes, eight orders, 37 families, 94 genera and 140 species were collected.

**Table:** Genera and Species

Genera and Species	Family	Order	Class
*Odontophora peritricha* Wieser, 1956	Axonolaimidae	Araeolaimida	Chromadorea
*Synodontium* sp. 1	Axonolaimidae	Araeolaimida	Chromadorea
*Comesoma* sp. 1	Comesomatidae	Araeolaimida	Chromadorea
*Metasabatieria* sp. 1	Comesomatidae	Araeolaimida	Chromadorea
*Sabatieria mortenseni* (Ditlevsen, 1921)	Comesomatidae	Araeolaimida	Chromadorea
*Sabatieria punctata* (Kreis, 1924)	Comesomatidae	Araeolaimida	Chromadorea
*Sabatieria wieseri* Platt, 1985	Comesomatidae	Araeolaimida	Chromadorea
*Coninckia* sp. 1	Coninckiidae	Araeolaimida	Chromadorea
*Campylaimus gerlachi* Timm, 1961	Diplopeltidae	Araeolaimida	Chromadorea
*Campylaimus* sp. 2	Diplopeltidae	Araeolaimida	Chromadorea
*Diplopeltula* sp.1	Diplopeltidae	Araeolaimida	Chromadorea
*Chromadora nudicapitata* Bastian, 1865	Chromadoridae	Chromadorida	Chromadorea
*Chromadorella* sp.1	Chromadoridae	Chromadorida	Chromadorea
*Chromadorina longispiculum* Pastor de Ward, 1985	Chromadoridae	Chromadorida	Chromadorea
*Prochromadorella* sp. 1	Chromadoridae	Chromadorida	Chromadorea
*Prochromadorella* sp. 2	Chromadoridae	Chromadorida	Chromadorea
*Actinonema* sp. 1	Chromadoridae	Chromadorida	Chromadorea
*Rhips* sp. 1	Chromadoridae	Chromadorida	Chromadorea
*Dichromadora* sp. 1	Chromadoridae	Chromadorida	Chromadorea
*Neochromadora alejandroi* Lo Russo & Pastor de Ward, 2012	Chromadoridae	Chromadorida	Chromadorea
*Neochromadora papillosa* Pastor de Ward, 1865	Chromadoridae	Chromadorida	Chromadorea
*Spilophorella paradoxa* (De Man, 1888)	Chromadoridae	Chromadorida	Chromadorea
*Marylynnia* sp. 1	Cyatholaimidae	Chromadorida	Chromadorea
*Marylynnia* sp. 2	Cyatholaimidae	Chromadorida	Chromadorea
*Marylynnia* sp. 3	Cyatholaimidae	Chromadorida	Chromadorea
*Paracanthonchus austrospectabilis* Wieser, 1954	Cyatholaimidae	Chromadorida	Chromadorea
*Paracanthonchus punctatus* (Bastian, 1865)	Cyatholaimidae	Chromadorida	Chromadorea
*Paracanthonchus* sp. 1	Cyatholaimidae	Chromadorida	Chromadorea
*Paracanthonchus* sp. 2	Cyatholaimidae	Chromadorida	Chromadorea
*Paracanthonchus* sp. 3	Cyatholaimidae	Chromadorida	Chromadorea
*Paracyatholaimus chilensis* Gerlach, 1953	Cyatholaimidae	Chromadorida	Chromadorea
*Paracyatholaimus* sp. 1	Cyatholaimidae	Chromadorida	Chromadorea
*Pomponema* sp. 1	Cyatholaimidae	Chromadorida	Chromadorea
*Paraethmolaimus dahli* (Gerlach, 1953)	Ethmolaimidae	Chromadorida	Chromadorea
*Gammanema* sp. 1	Selachinematidae	Chromadorida	Chromadorea
*Gammanema* sp. 2	Selachinematidae	Chromadorida	Chromadorea
*Halichoanolaimus* sp. 1	Selachinematidae	Chromadorida	Chromadorea
*Latronema* sp. 1	Selachinematidae	Chromadorida	Chromadorea
*Molgolaimus* sp. 1	Desmodoridae	Desmodorida	Chromadorea
*Molgolaimus* sp. 2	Desmodoridae	Desmodorida	Chromadorea
*Metachromadora* sp. 1	Desmodoridae	Desmodorida	Chromadorea
*Metachromadora spectans* Gerlach, 1957	Desmodoridae	Desmodorida	Chromadorea
*Onyx* sp. 1	Desmodoridae	Desmodorida	Chromadorea
*Polysigma* sp. 1	Desmodoridae	Desmodorida	Chromadorea
*Spirinia septentrionalis* Cobb, 1914	Desmodoridae	Desmodorida	Chromadorea
*Bolbolaimus* sp. 2	Microlaimidae	Desmodorida	Chromadorea
*Microlaimus conothelis* (Lorenzen, 1973) Jensen, 1978	Microlaimidae	Desmodorida	Chromadorea
*Microlaimus capillaris* Gerlach, 1957	Microlaimidae	Desmodorida	Chromadorea
*Microlaimus decoratus* Pastor de Ward, 1991	Microlaimidae	Desmodorida	Chromadorea
*Microlaimus globiceps* De Man, 1880	Microlaimidae	Desmodorida	Chromadorea
*Microlaimus* sp. 2	Microlaimidae	Desmodorida	Chromadorea
*Nudora besnardi* (Gerlach, 1956)	Monoposthiidae	Desmodorida	Chromadorea
*Nudora crepidata* Wieser, 1954	Monoposthiidae	Desmodorida	Chromadorea
*Desmolaimus* sp. 3	Linhomoeidae	Monhysterida	Chromadorea
*Desmolaimus* sp. 4	Linhomoeidae	Monhysterida	Chromadorea
*Metalinhomoeus gloriae* Pastor de Ward, 1989	Linhomoeidae	Monhysterida	Chromadorea
*Metalinhomoeus parafiliformis* Pastor de Ward, 1989	Linhomoeidae	Monhysterida	Chromadorea
*Metalinhomoeus typicus* De Man, 1907	Linhomoeidae	Monhysterida	Chromadorea
*Terschellingia longicaudata* De Man, 1907	Linhomoeidae	Monhysterida	Chromadorea
*Terschellingia* sp. 2	Linhomoeidae	Monhysterida	Chromadorea
*Eleutherolaimus* sp. 1	Linhomoeidae	Monhysterida	Chromadorea
*Paralinhomoeus visitus* Pastor de Ward, 1989	Linhomoeidae	Monhysterida	Chromadorea
*Siphonolaimus auratus* Wieser, 1956	Siphonolaimidae	Monhysterida	Chromadorea
*Diplolaimella gerlachi* Pastor de Ward, 1984	Monhysteridae	Monhysterida	Chromadorea
*Diplolaimella ocellata* (Bütschli, 1874)	Monhysteridae	Monhysterida	Chromadorea
*Diplolaimelloides oschei* Meyl, 1954	Monhysteridae	Monhysterida	Chromadorea
*Halomonhystera disjuncta* (Bastian, 1865)	Monhysteridae	Monhysterida	Chromadorea
*Thalassomonhystera parva* (Bastian, 1865)	Monhysteridae	Monhysterida	Chromadorea
*Sphaerolaimus pacificus* Allgen, 1945	Sphaerolaimidae	Monhysterida	Chromadorea
*Amphimonhystera* sp. 2	Xyalidae	Monhysterida	Chromadorea
*Cobbia macrodentata* Lo Russo & Pastor de Ward, 2012	Xyalidae	Monhysterida	Chromadorea
*Daptonema laxus* Wieser, 1956	Xyalidae	Monhysterida	Chromadorea
*Daptonema* sp. 2	Xyalidae	Monhysterida	Chromadorea
*Daptonema* sp. 3	Xyalidae	Monhysterida	Chromadorea
*Gonionchus* sp. 1	Xyalidae	Monhysterida	Chromadorea
*Linhystera* sp. 1	Xyalidae	Monhysterida	Chromadorea
*Metadesmolaimus* sp. 3	Xyalidae	Monhysterida	Chromadorea
*Metadesmolaimus* sp. 4	Xyalidae	Monhysterida	Chromadorea
*Metadesmolaimus* sp. 5	Xyalidae	Monhysterida	Chromadorea
*Omicronema* sp. 1	Xyalidae	Monhysterida	Chromadorea
*Paramonohystera* sp. 4	Xyalidae	Monhysterida	Chromadorea
*Promonhystera* sp. 1	Xyalidae	Monhysterida	Chromadorea
*Pseudosteineria* sp. 1	Xyalidae	Monhysterida	Chromadorea
*Rhynchonema separatum* Lorenzen, 1975	Xyalidae	Monhysterida	Chromadorea
*Rhynchonema* sp. 1	Xyalidae	Monhysterida	Chromadorea
*Rhynchonema* sp. 2	Xyalidae	Monhysterida	Chromadorea
*Rhynchonema* sp. 3	Xyalidae	Monhysterida	Chromadorea
*Theristus lorenzeni* Pastor de Ward, 1985	Xyalidae	Monhysterida	Chromadorea
*Theristus modicus* Wieser, 1956	Xyalidae	Monhysterida	Chromadorea
*Theristus* sp. 2	Xyalidae	Monhysterida	Chromadorea
*Theristus* sp. 3	Xyalidae	Monhysterida	Chromadorea
*Theristus* sp. 4	Xyalidae	Monhysterida	Chromadorea
*Theristus* sp. 5	Xyalidae	Monhysterida	Chromadorea
*Xyalidae* gen. 1 sp. 1	Xyalidae	Monhysterida	Chromadorea
*Ceramonema* sp. 1	Ceramonematidae	Plectida	Chromadorea
*Pselionema* sp. 1	Ceramonematidae	Plectida	Chromadorea
*Tarvaia* sp. 1	Tarvaiidae	Plectida	Chromadorea
*Haliplectus salicornius* Pastor de Ward, 1984	Haliplectidae	Plectida	Chromadorea
*Cyartonema flexile* Cobb, 1920	Aegialoalaimidae	Plectida	Chromadorea
*Cyartonema* sp. 1	Aegialoalaimidae	Plectida	Chromadorea
*Deontolaimus papillatus* De Man, 1880	Leptolaimidae	Plectida	Chromadorea
*Leptolaimus luridus* Timm, 1963	Leptolaimidae	Plectida	Chromadorea
*Leptolaimus puccinelliae* Gerlach, 1959	Leptolaimidae	Plectida	Chromadorea
*Mesorhabditis* sp. 2	Mesorhabditidae	Rhabditida	Chromadorea
*Macroposthonia* sp. 1	Criconematidae	Rhabditida	Chromadorea
*Panagrolaimus* sp. 1	Panagrolaimidae	Rhabditida	Chromadorea
*Tylenchus* sp. 1	Tylenchidae	Rhabditida	Chromadorea
*Dorylaimus* sp. 1	Dorylaimidae	Dorylaimida	Enoplea
*Chaetonema patagonica* Lo Russo et al., 2015	Anoplostomatidae	Enoplida	Enoplea
*Cephalanticoma* sp. 1	Anticomidae	Enoplida	Enoplea
*Enoplus benhami* Ditlevsen, 1930	Enoplidae	Enoplida	Enoplea
*Enoplus meridionalis* Steiner, 1921	Enoplidae	Enoplida	Enoplea
*Epacanthion bicuspidatum* Lo Russo et al., 2012	Thoracostomopsidae	Enoplida	Enoplea
*Oxyonchus* sp. 1	Thoracostomopsidae	Enoplida	Enoplea
*Thoracostomopsidae* gen. 1 sp. 2	Thoracostomopsidae	Enoplida	Enoplea
*Conilia divina* Gerlach, 1956	Ironoidae	Enoplida	Enoplea
*Dolicholaimus marioni* De Man, 1888	Ironoidae	Enoplida	Enoplea
*Syringolaimus smarigdus* Cobb, 1928	Ironoidae	Enoplida	Enoplea
*Halalaimus* sp. 1	Oxystominidae	Enoplida	Enoplea
*Halalaimus* sp. 2	Oxystominidae	Enoplida	Enoplea
*Halalaimus* sp. 3	Oxystominidae	Enoplida	Enoplea
*Halalaimus* sp. 4	Oxystominidae	Enoplida	Enoplea
*Thalassoalaimus* sp. 2	Oxystominidae	Enoplida	Enoplea
*Calyptronema keiense* Wieser 1953	Enchelidiidae	Enoplida	Enoplea
*Calyptronema maxweberi* (De Man, 1922)	Enchelidiidae	Enoplida	Enoplea
*Eurystomina* sp. 1	Enchelidiidae	Enoplida	Enoplea
*Abelbolla* sp. 1	Enchelidiidae	Enoplida	Enoplea
*Adoncholaimus* sp. 2	Oncholaimidae	Enoplida	Enoplea
*Oncholaimellus paracarlbergi* Pastor de Ward, 1993	Oncholaimidae	Enoplida	Enoplea
*Viscosia macramphida* Chitwood, 1951	Oncholaimidae	Enoplida	Enoplea
*Metoncholaimus* sp. 1	Oncholaimidae	Enoplida	Enoplea
*Metoncholaimus* sp. 2	Oncholaimidae	Enoplida	Enoplea
*Oncholaimus* sp. 1	Oncholaimidae	Enoplida	Enoplea
*Oncholaimus* sp. 2	Oncholaimidae	Enoplida	Enoplea
*Lauratonema* sp. 1	Lauratonematidae	Enoplida	Enoplea
*Rhabdocoma* sp. 1	Trefusiidae	Enoplida	Enoplea
*Trefusia litoralis* (Allgén, 1932)	Trefusiidae	Enoplida	Enoplea
*Trefusiidae* gen. 1 sp. 1	Trefusiidae	Enoplida	Enoplea
*Bathylaimus australis* Cobb, 1894	Tripyloididae	Enoplida	Enoplea
*Tripyloides amazonicus* (Gerlach, 1957)	Tripyloididae	Enoplida	Enoplea

**Quality control description**: The geo-referencing of all specimens were recorded using a Garmin eTrex Legend GPS (WGS84 Datum) with an accuracy of less than 10 m and with at least 5 satellites. The taxonomic identification of specimens, scientific names, and their current accurate spelling were verified by C. Pastor de Ward, a free-living marine nematode specialist. Other post-validation procedures (including geographic coordinate format, congruence between collection and identification dates, absence of ASCII anomalous characters) were checked using the Darwin Test software (http://www.gbif.es/darwin_test/Darwin_Test_in.php).

## Dataset description

**Object name**: Darwin Core Archive free-living marine Nematodes from San Antonio Bay (Río Negro, Argentina).

**Character encoding**: UTF-8

**Format name**: Darwin Core Archive format

**Format version**: 1.0

**Distribution**: http://ipt.cenpat-conicet.gob.ar:8081/resource?r=sao2009#

**Publication date of data**: 2013-10-25

**Language**: English

**Licenses of use**: This work is licensed under a Creative Commons CC0 1.0 License http://creativecommons.org/publicdomain/zero/1.0/legalcode

**External datasets**

**Object name**: Centro Nacional Patagónico (CENPAT-CONICET)

**Distribution**: http://ipt.cenpat-conicet.gob.ar:8081/resource?r=sao2009#

**Object name**: Ministerio de Ciencia y Tecnología de Argentina (Sistema Nacional de Datos Biológicos - SNDB)

**Distribution**: GBIF: http://www.gbif.org/dataset/d592283b-b00e-4a39-9499-289842ccddf1

**Formatted**: English (U.K.)

**Field Code Changed**

**Metadata language**: English

**Date of metadata creation**: 2014-08-27

**Hierarchy level**: Dataset

## References

[B1] Abebe E Andrássy I Traunspurger W (2006) Freshwater nematodes: ecology and taxonomy. CABI Publishing, Wallingford, United Kingdom, 752 pp.

[B2] De Ley P Blaxter M (2004) A new system for Nematoda: combining morphological characters with molecular trees, and translating clades into ranks and taxa. Nematology Monographs and Perspectives 2: 633–653.

[B3] Lorenzen S (1994) The Phylogenetic Systematics of Free-living Nematodes. The Ray Society, Surrey, United Kingdom, 383 pp.

[B4] Lo Russo V (2012) Comparación de comunidades de nematodos de marismas de San Antonio Oeste (Río Negro) y San Julián (Sta. Cruz). Doctoral thesis, Universidad Nacional del COMAHUE, 214 pp.

[B5] Lo Russo V Pastor de Ward CT (2012) *Neochromadora alejandroi* sp. n. (Chromadorida: Chromadoridae) and *Cobbia macrodentata* sp. n. (Monhysterida: Xyalidae), two new species of free-living marine nematodes from the Patagonian coast. Nematology 14(7): 805–815. doi: 10.1163/145854112X627327

[B6] Lo Russo V Villares G Martelli A Pastor de Ward CT Harguinteguy C (2012) New species of *Epacanthion* (Nematoda: Thoracostomopsidae) from Patagonia coast, Río Negro and Chubut, Argentina. Journal of the Marine Biological Association of the United Kingdom 93: 925–934. doi: 10.1017/S002531541200080X

[B7] Lo Russo V Villares G Pastor de Ward CT (2015) New species of *Chaetonema* (Nematoda, Anoplostomatidae) and *Admirandus* (Nematoda, Oncholaimidae) from Patagonia, Río Negro and Santa Cruz, Argentina. Journal of the Marine Biological Association of the United Kingdom. doi: 10.1017/S0025315415002039

[B8] Pastor de Ward CT (1978) Free-living marine nematodes (Subclass Adenophorea) of the ría Deseado (Santa Cruz, Argentina). Systematic Contributions I. Annales de la Societe Royale Zoologique de Belgique 1-2: 29–45.

[B9] Pastor de Ward CT (1980) *Aponema papillatum* sp. nov. nueva especie de nematodo marino de Puerto Deseado, Argentina. Contribuciones Científicas del CIBIMA Nro. 160: 3–11.

[B10] Pastor de Ward CT (1984a) Tres especies nuevas de nematodos marinos de vida libre (Chromadoridae y Comesomatidae) para la ría Deseado, Santa Cruz, Argentina. Physis A 42: 39–48.

[B11] Pastor de Ward CT (1984b) Nematodos marinos de la ría Deseado (Leptolaimina: Leptolaimidae, Haliplectidae) Santa Cruz, Argentina. I. Physis A 42: 87–92.

[B12] Pastor de Ward CT (1984c) Nematodos marinos de la ría Deseado (Monhysteroidea: Sphaerolaimidae, Monhysteridae), Santa Cruz, Argentina. 3. Contribuciones del Centro Nacional Patagónico Nro. 85: 1–15.

[B13] Pastor de Ward CT (1984d) Nematodos marinos de la ría Deseado (Axonolaimoidea: Axonolaimidae, Diplopeltidae, Comesomatidae) Santa Cruz, Argentina. 4. Contribuciones del Centro Nacional Patagónico Nro. 86: 1–21.

[B14] Pastor de Ward CT (1984e) *Ptycholaimellus setosus* sp. nov., nueva especie de nematodo marino de vida libre (Chromadoridae, Hypodontolaiminae) de Puerto Deseado, Santa Cruz, Argentina. Neotropica 30: 11–18.

[B15] Pastor de Ward CT (1985) Nematodos marinos de la ría Deseado (Monhysteroidea: Xyalidae), Santa Cruz, Argentina, 2. Physis A 43: 113–130.

[B16] Pastor de Ward CT (1986) Free-living marine nematodes of the Deseado river estuary (Chromadoroidea: Chromadoridae, Ethmolaimidae, Cyatholaimidae and Choniolaimidae) Santa Cruz, Argentina. 5. Publicación Especial del Centro Nacional Patagónico Nro. 6: 1–83.

[B17] Pastor de Ward CT (1988) Nematodos marinos de la ría Deseado (Desmodoroidea: Desmodoridae, Draconematidae) Santa Cruz, Argentina 7. Physis A 46: 61–72.

[B18] Pastor de Ward CT (1989) Free-living marine nematodes of the Deseado River estuary (Siphonolaimoidea: Siphonolaimidae, Linhomoeidae) Santa Cruz, Argentina, 6. Studies on Neotropical Fauna and Environment 24: 231–247. doi: 10.1080/01650528909360794

[B19] Pastor de Ward CT (1990) Nematodos marinos de la ría Deseado (Oncholaimoidea: Oncholaimidae), Santa Cruz, Argentina, 10. Physis A 48: 29–40.

[B20] Pastor de Ward CT (1991) Nematodos marinos de la ría Deseado (Oncholaimoidea: Enchelidiidae), Santa Cruz, Argentina, 11. Physis A 49: 27–39.

[B21] Pastor de Ward CT (1991) Nematodos marinos de la ría Deseado (Microlaimoidea: Microlaimidae, Monoposthiidae), Santa Cruz, Argentina, 8. Physis A 47: 1–12.

[B22] Pastor de Ward CT (1993) Nematodos marinos de vida libre de la ría Deseado (Tripyloidina, Tripyloididae), Santa Cruz, Argentina. 13. Naturalia Patagónica 1: 61–67.

[B23] Pastor de Ward CT (1995a) Free-living marine nematodes from Deseado river estuary (Ironoidea: Leptosomatidae, Thoracostomatidae). Santa Cruz, Argentina. 12. Spixiana 18: 201–209.

[B24] Pastor de Ward CT (1995b) Nematodos marinos de la ría Deseado (Enoploidea), Santa Cruz, Argentina. 14. Physis A 50: 13–20.

[B25] Pastor de Ward CT (1996) *Deontostoma* species from subantarctic coasts (Nematoda, Leptosomatidae). Hidrobiologia 315: 177–187. doi: 10.1007/BF00051948

[B26] Pastor de Ward CT (1998a) Distribución espacial de nematodos libres de la ría Deseado, Santa Cruz (Patagonia, Argentina). Revista de Biología Marina y Oceanografía 33: .

[B27] Pastor de Ward CT (1998b) New free-living marine nematodos from Deseado river estuary (Ironoidea: Oxystominidae) Santa Cruz, Argentina. XV. Physis A 56: 1–6.

[B28] Pastor de Ward CT (1998c) Nematodos marinos de la ría Deseado (Desmodoroidea: Desmodoridae, Draconematidae), Santa Cruz, Argentina. VII. Physis A 46: 61–72.

[B29] Pastor de Ward CT (1999) The structure of the genital apparatus in Nematoda (Chromadoroidea) from South Atlanctic Coasts. Physis A 57: 47–54.

[B30] Pastor de Ward CT Lo Russo V (2009) Distribution of *Diplolaimella* and *Diplolaimelloides* species from Patagonian lagoons and coastal waters (Nematoda: Monhysteridae), Chubut and Santa Cruz provinces. Journal of the Marine Biological Association of the United Kingdom 89: 711–718. doi: 10.1017/S0025315409000198

[B31] Pastor de Ward CT Lo Russo V Villares G Martelli A (2013) Three new species of the free-living nematode genus *Oncholaimus* Dujardin, 1845 (Enoplida, Oncholaimidae) from Atlantic coasts of Argentina. Journal of Morphology and Systematics (Jaen) 16: 131–141.

[B32] Platt H Warwick RM (1983) Free-living Marine Nematodes. Part I. British Enoplids. Synopses of British Fauna (New Series) N°28, Barnes, Crothers, Cambridge University Press, 307 pp.

[B33] Platt H Warwick RM (1988) Free-living Marine Nematodes. Part II. British Chromadorids. Synopses of British Fauna (New Series) N°38, Barnes, Crothers, Cambridge University Press, 499 pp.

[B34] Somerfield PJ Warwick RM (1996) Meiofauna in marine pollution monitoring programmes. A laboratory manual. Ministery of Agriculture, Fisheries and Food, Directorate of Fisheries Research, Lowestoft, 71 pp.

[B35] Villares G Pastor de Ward CT (2012) New species of *Antomicron* and *Leptolaimus* (Nematoda: Leptolaimidae) and record of *Procamacolaimus* (Nematoda: Camacolaimidae) from Patagonia coast, Chubut and Santa Cruz, Argentina. Journal of the Marine Biological Association of the United Kingdom 92: 929–939. doi: 10.1017/S0025315411000269

[B36] Villares G Martelli A Lo Russo V Pastor de Ward CT (2013) Three new species and one new record of *Campylaimus* (Diplopeltidae, Nematoda) from Argentine coasts (Buenos Aires and Santa Cruz, Argentina). Zootaxa 3613: 083–096. doi: 10.11646/zootaxa.3613.1.4 10.11646/zootaxa.3613.1.4 24698903

[B37] Villares G (2014) Diversidad funcional y producción secundaria de las comunidades de nematodos de las marismas de San Antonio (Río Negro) y de la ría de San Julián (Santa Cruz). Doctoral thesis, Universidad Nacional del COMAHUE, 144 pp.

[B38] Warwick RM Platt H Somerfield PJ (1998) Free-living Marine Nematodes. Part III. Monhysterids. Synopses of British Fauna (New Series) N°53, Barnes, Crothers, Cambridge University Press, 296 pp.

